# Treated, hospital-onset *Clostridiodes difficile* infection: An evaluation of predictors and feasibility of benchmarking comparing 2 risk-adjusted models among 265 hospitals

**DOI:** 10.1017/ice.2023.124

**Published:** 2024-01

**Authors:** Kalvin C. Yu, Gang Ye, Jonathan R. Edwards, Raymund Dantes, Vikas Gupta, ChinEn Ai, Kristina Betz, Andrea L. Benin

**Affiliations:** 1 Becton, Dickinson and Company, Franklin Lakes, New Jersey; 2 Centers for Disease Control and Prevention, Atlanta, Georgia; 3 Emory University School of Medicine, Atlanta, Georgia

## Abstract

**Objectives:**

To evaluate the incidence of a candidate definition of healthcare facility-onset, treated *Clostridioides difficile* (CD) infection (cHT-CDI) and to identify variables and best model fit of a risk-adjusted cHT-CDI metric using extractable electronic heath data.

**Methods:**

We analyzed 9,134,276 admissions from 265 hospitals during 2015–2020. The cHT-CDI events were defined based on the first positive laboratory final identification of CD after day 3 of hospitalization, accompanied by use of a CD drug. The generalized linear model method via negative binomial regression was used to identify predictors. Standardized infection ratios (SIRs) were calculated based on 2 risk-adjusted models: a simple model using descriptive variables and a complex model using descriptive variables and CD testing practices. The performance of each model was compared against cHT-CDI unadjusted rates.

**Results:**

The median rate of cHT-CDI events per 100 admissions was 0.134 (interquartile range, 0.023–0.243). Hospital variables associated with cHT-CDI included the following: higher community-onset CDI (CO-CDI) prevalence; highest-quartile length of stay; bed size; percentage of male patients; teaching hospitals; increased CD testing intensity; and CD testing prevalence. The complex model demonstrated better model performance and identified the most influential predictors: hospital-onset testing intensity and prevalence, CO-CDI rate, and community-onset testing intensity (negative correlation). Moreover, 78% of the hospitals ranked in the highest quartile based on raw rate shifted to lower percentiles when we applied the SIR from the complex model.

**Conclusions:**

Hospital descriptors, aggregate patient characteristics, CO-CDI burden, and clinical testing practices significantly influence incidence of cHT-CDI. Benchmarking a cHT-CDI metric is feasible and should include facility and clinical variables.

*Clostridioides difficile* (CD) infection (CDI) remains one of the most commonly occurring healthcare-associated infections (HAIs). CDI is associated with 15,000–30,000 annual deaths and excess inpatient costs exceeding $4.8 billion in the United States.^1–5^ CDI affects 14 in every 1,000 patients^5^; ∼24%–70% of cases are classified as hospital-onset (HO) CDI (HO-CDI) depending on the definition applied.^3,6^ Since the application of polymerase chain reaction (PCR) technology, practices for testing and treatment of CDI have evolved. CD PCR testing alone may be hypersensitive,^7^ and current approaches to HO-CDI testing often involve multistep algorithms to help improve specificity of testing and treatment.^1^ Unfortunately, the risk of overdiagnosis along with the additional cost and resources of tiered testing have complicated feasibility and best-practice standards.^8–10^ The heterogeneous approaches to diagnostic testing have led to varied sensitivity and specificity levels for laboratory-based identification and surveillance. Furthermore, CDI is most appropriately considered a clinical diagnosis that typically involves the aggregate evaluation of laboratory identification, clinical history, and physical exam.^11,12^

The Centers for Disease Control and Prevention (CDC) National Healthcare Safety Network (NHSN) has facilitated CDI surveillance using a definition based only on laboratory identification. However, an opportunity may exist to more accurately define CDI by combining laboratory identification with additional data sources accessible in electronic health records (EHR). We evaluated the incidence of a candidate definition for healthcare facility-onset, treated CDI (cHT-CDI). We identified hospital-level variables associated with incidence and developed a risk-adjusted cHT-CDI metric using data that could be easily extracted from EHR. Given the complexities of current CD testing and the clinical judgement required to diagnose and treat CDI, we developed a definition of cHT-CDI that simultaneously included a laboratory identification of CD accompanied by administration of an anti-CD antimicrobial, which served as a proxy for clinical interpretation of infection. We then used this candidate definition to evaluate cHT-CDI incidence and to identify hospital-level variables using statistical models to help assess a standardized cHT-CDI risk-adjusted metric. Standardized infection ratios (SIRs), defined as the ratio of observed number of events (cHT-CDI) divided by the predicted (expected) number of events, were calculated based on a simple model regression analysis (using EHR-extractable risk factors as variables) and a complex model (which additionally included CD testing practice variables), an approach that has been used successfully for hospital-onset bacteremia.^13^ We compared hospital rankings based on the unadjusted rate of cHT-CDI and on the simple and complex model–based SIRs.

## Methods

### Study design and population

This study was a retrospective, ecological study based on electronic microbiological, medication, and administrative data within the BD Insights and Research Database (Becton Dickinson, Franklin Lakes, NJ) from adult patients aged ≥18 years admitted to 1 of 265 acute-care hospitals between October 1, 2015, and February 28, 2020.^14–16^ The study used a limited retrospective data set for an epidemiology study and thus was approved and exempted from consent by the New England Institutional Review Board/Human Subjects Research Committee (Wellesley, MA). The study was conducted in compliance with Health Insurance Portability and Accountability Act requirements.

### Outcomes and definitions

The date of admission (day the patient’s status changed to inpatient) was considered day 1. A cHT-CDI event was defined when the following 2 requirements were met: (1) a final report by the facility laboratory of CD positive (ie, positive result of a single test for facilities relying on single tests or positive confirmation test in facilities with multitest rubrics) within the HO period (ie, day 4 or greater of hospitalization) and (2) receipt of at least 1 anti-CD antimicrobial (ie, oral vancomycin, fidaxomicin, or oral metronidazole) with a first qualified antimicrobial day (QAD) in the window period extending 2 calendar days before and 2 calendar days after the positive CD specimen-collection date. This QAD definition is similar to the one used in the CDC Hospital Toolkit for Adult Sepsis Surveillance.^17^ Community-onset (CO) CDI (CO-CDI) was defined as a first positive CD test within the first 3 days of hospital admission. Patients with a positive CD test in our database within 4 weeks prior to study admission were excluded. Subsequent duplicate tests from a CD-positive patient during the study were also excluded.

Testing intensity was defined as the number of total stool specimens tested for CD in the CO or HO periods divided by the number of total aggregate admissions with any CD test performed. Conceptually, testing intensity reflects the cumulative CD-tested stool samples collected among admissions with any CD test performed. Testing prevalence was defined as the number of admissions with any CD test performed in the period (CO or HO) divided by the total number of aggregate admissions and conceptually reflects the overall proportion of admissions with CD testing.

### Statistical analysis

We approached the statistical analysis in 3 steps: (1) identify candidate variables that influence cHT-CDI rates using bivariate analysis; (2) construct simple and complex models to predict cHT-CDI using risk-adjusted SIRs derived from regression models and assess best model fit; and (3) compare hospital rankings using the observed (unadjusted) cHT-CDI rates versus rankings based on SIRs derived from the 2 models.

Step 1. Rates of cHT-CDI were calculated as the number of cHT-CDI events per 100 admissions for quarterly aggregated data. A bivariate analysis using generalized linear models was performed to explore the correlation between cHT-CDI rate and each candidate risk factor (Table 1). The following clinical metrics were measured: CO-CDI prevalence (rate of CO-CDI events per 100 admissions); percentage of intensive care unit (ICU) admissions (per all admissions); average length of hospital stay (LOS) among hospitalized patients (days of hospitalization per admission); CD test prevalence and intensity (as previously defined). We also collected the following patient demographics: number of females per 100 admissions; percentages of patients in each age group (ie, 18–40, 41–64, 65–80, and >80 years). In addition, facility characteristics included bed size, medical school affiliation, urban or rural status.

**Table 1.** Descriptive Statistics of cHT-CDI Rate and Bivariate Analysis Results

Variables	Admissions	cHT-CDI Events	Observed cHT-CDI Rate per 100 Admissions					
			Lower Quartile	Median	Upper Quartile	Mean	SD	*P* Value
Overall	9,134,276	17,545	0.023	0.134	0.243	0.166	0.180	
**Year**								<.0001
2015	344,056	859	0.080	0.180	0.293	0.221	0.252	
2016	1,714,354	4,083	0.071	0.171	0.295	0.202	0.196	
2017	2,042,560	4,445	0.063	0.167	0.286	0.206	0.208	
2018	2,305,170	4,273	0.000	0.132	0.240	0.159	0.169	
2019	2,226,722	3,171	0.000	0.096	0.191	0.123	0.137	
2020	501,414	714	0.000	0.096	0.172	0.121	0.122	
**CO-CDI rate per 100 admissions**								<.0001
1st quartile	1,686,392	1,540	0.000	0.000	0.110	0.070	0.122	
2nd quartile	3,056,765	4,944	0.055	0.129	0.211	0.142	0.112	
3rd quartile	2,496,840	5,342	0.089	0.171	0.268	0.188	0.149	
4th quartile	1,894,279	5,719	0.102	0.225	0.369	0.264	0.244	
**Average LOS**								<.0001
1st quartile	977,467	1,158	0.000	0.053	0.159	0.098	0.133	
2nd quartile	2,206,252	3,467	0.039	0.126	0.228	0.156	0.170	
3rd quartile	2,800,069	5,248	0.074	0.158	0.268	0.179	0.139	
4th quartile	3,150,488	7,672	0.084	0.198	0.311	0.232	0.232	
**% ICU admissions**								0.0493
Not reported	385,270	1,015	0.000	0.082	0.232	0.186	0.332	
1st quartile	1,622,463	2,859	0.000	0.087	0.199	0.131	0.173	
2nd quartile	2,611,370	4,219	0.059	0.136	0.239	0.162	0.137	
3rd quartile	2,273,673	4,590	0.064	0.158	0.266	0.182	0.158	
4th quartile	2,241,500	4,862	0.039	0.163	0.270	0.183	0.164	
**Testing prevalence**								<.0001
1st quartile	1,905,258	1,988	0.000	0.028	0.123	0.076	0.110	
2nd quartile	2,804,156	4,385	0.051	0.126	0.199	0.135	0.122	
3rd quartile	2,473,500	4,978	0.075	0.164	0.264	0.175	0.130	
4th quartile	1,951,362	6,194	0.114	0.245	0.389	0.278	0.252	
**CO testing prevalence**								<.0001
1st quartile	2,257,847	2,925	0.000	0.064	0.158	0.101	0.136	
2nd quartile	3,000,705	5,276	0.051	0.135	0.217	0.147	0.122	
3rd quartile	2,190,180	4,396	0.068	0.162	0.263	0.178	0.140	
4th quartile	1,685,544	4,948	0.050	0.185	0.342	0.238	0.257	
**HO testing prevalence**								<.0001
1st quartile	1,457,185	1,184	0.000	0.000	0.082	0.052	0.086	
2nd quartile	2,259,355	2,901	0.039	0.112	0.174	0.118	0.107	
3rd quartile	2,680,913	5,117	0.097	0.178	0.250	0.182	0.130	
4th quartile	2,736,823	8,343	0.171	0.279	0.399	0.312	0.237	
**Testing intensity**								<.0001
Not calculated^a^	8,663	0	0.000	0.000	0.000	0.000	0.000	
1st quartile	1,715,666	2,630	0.000	0.083	0.193	0.120	0.156	
2nd quartile	2,362,434	4,778	0.061	0.157	0.249	0.172	0.141	
3rd quartile	2,803,869	5,997	0.071	0.169	0.295	0.203	0.188	
4th quartile	2,243,644	4,140	0.041	0.131	0.247	0.179	0.215	
**CO testing intensity**								<.0001
Not calculated^a^	8,663	0	0.000	0.000	0.000	0.000	0.000	
1st quartile	2,823,284	6,946	0.086	0.194	0.309	0.232	0.238	
2nd quartile	2,479,886	4,855	0.077	0.169	0.267	0.185	0.146	
3rd quartile	2,117,783	3,527	0.039	0.135	0.239	0.158	0.149	
4th quartile	1,704,660	2,217	0.000	0.056	0.150	0.098	0.140	
**HO testing intensity**								<.0001
Not calculated^a^	8,663	0	0.000	0.000	0.000	0.000	0.000	
1st quartile	1,166,641	1,101	0.000	0.000	0.117	0.070	0.106	
2nd quartile	2,101,608	3,320	0.055	0.132	0.222	0.155	0.135	
3rd quartile	2,754,801	5,432	0.088	0.177	0.281	0.199	0.164	
4th quartile	3,102,563	7,692	0.109	0.207	0.332	0.250	0.236	
**% Female**								<.0001
1st quartile	2,553,168	5,532	0.045	0.161	0.266	0.194	0.217	
2nd quartile	2,741,130	5,253	0.055	0.147	0.256	0.172	0.156	
3rd quartile	2,314,064	4,320	0.038	0.137	0.250	0.173	0.184	
4th quartile	1,525,914	2,440	0.000	0.086	0.204	0.126	0.148	
**% Patients aged 18–40 y**								<.0001
1st quartile	1,430,875	3,056	0.000	0.093	0.243	0.162	0.243	
2nd quartile	2,489,638	4,686	0.061	0.156	0.260	0.183	0.163	
3rd quartile	3,024,935	5,738	0.068	0.144	0.249	0.166	0.138	
4th quartile	2,188,828	4,065	0.000	0.128	0.228	0.154	0.156	
**% Patients aged 41–64 y**								<.0001
1st quartile	1,478,674	2,405	0.000	0.095	0.209	0.136	0.172	
2nd quartile	2,313,483	4,110	0.050	0.145	0.252	0.171	0.161	
3rd quartile	2,376,773	4,592	0.042	0.138	0.249	0.166	0.152	
4th quartile	2,965,346	6,438	0.053	0.150	0.261	0.192	0.221	
**% Patients aged 65–80 y**								0.066
1st quartile	2,307,177	4,508	0.000	0.130	0.229	0.155	0.157	
2nd quartile	2,589,933	5,062	0.068	0.154	0.263	0.176	0.156	
3rd quartile	2,615,879	4,658	0.049	0.141	0.238	0.166	0.156	
4th quartile	1,621,287	3,317	0.000	0.103	0.253	0.168	0.237	
**% Patients aged >80 y**								0.0647
1st quartile	3,007,521	6,362	0.058	0.146	0.244	0.184	0.212	
2nd quartile	2,472,873	4,352	0.043	0.136	0.240	0.160	0.144	
3rd quartile	2,213,688	3,969	0.028	0.136	0.245	0.163	0.158	
4th quartile	1,440,194	2,862	0.000	0.120	0.243	0.158	0.195	
**Bed size**								<.0001
<100	587,288	763	0.000	0.000	0.154	0.101	0.189	
100–300	3,331,644	6,002	0.060	0.147	0.255	0.182	0.186	
>300	5,215,344	10,780	0.113	0.183	0.279	0.208	0.136	
**Bed size (refined grouping)**								<.0001
1–50	147,391	108	0.000	0.000	0.000	0.058	0.171	
51–100	439,897	655	0.000	0.096	0.222	0.144	0.197	
101–200	1,352,638	2,070	0.044	0.126	0.236	0.158	0.153	
201–300	1,979,006	3,932	0.089	0.169	0.281	0.212	0.217	
301–500	3,036,288	6,167	0.099	0.174	0.275	0.205	0.146	
500+	2,179,056	4,613	0.133	0.202	0.281	0.214	0.110	
**Teaching status (medical school affiliation)**								<.0001
Nonteaching	3,225,702	5,197	0.000	0.093	0.214	0.135	0.167	
Teaching	5,908,574	12,348	0.095	0.173	0.283	0.209	0.187	
**Urban–rural status**								0.013
Rural	2,391,775	4,397	0.000	0.096	0.228	0.144	0.177	
Urban	6,742,501	13,148	0.057	0.151	0.251	0.178	0.180	

Note. CO, community-onset; CO-CDI, community-onset *C. difficile* infection; HO, hospital-onset; cHT-CDI, candidate definition for healthcare facility-onset, treated *C. difficile* infection; ICU, intensive care unit; LOS, length of stay; SD, standard deviation.

a Due to zero denominator.

Step 2. To identify cHT-CDI-associated variables, we used negative binomial regression methods with natural logarithm link function to account for overdispersion and right-skewed data. We conducted 2 modeling analyses: a simple model and a complex model. The simple model used hospital-level variables easily accrued from EHRs and/or already reportable to the NHSN. Candidate variables considered in the simple model included hospital-level characteristics and patient demographics. Our complex model included variables considered in the simple model (but partitioned into quartiles) plus clinical practices of overall CD testing and divided into CO or HO testing intensity and prevalence.

The best models were selected using model fit statistics (ie, Akaike information criteria and Bayesian information criteria) based on the full study cohort data (3,455 quarters of aggregated hospital data associated with 9,134,276 admissions). In addition, we used cross-validation methods in variable selection and confirmed that the full-data model and validation model included the set of variables identical to the best models. Residual (prediction error) diagnosis of the best (complex) model was performed using decile plot, observed versus predicted cHT-CDI events plot, and standardized deviance residuals versus predicted events (Supplementary Figs. S1 and S2 online).

Step 3. We compared hospital rankings based on the observed (unadjusted) cHT-CDI rate and the ranking based on the SIRs from the simple and complex models. The Goodman and Kruskal γ statistic, Spearman correlation, and confidence intervals to measure ordinal association were calculated. We calculated 1-year SIR data (2019) as an example for comparison rankings. Finally, we compared the ranking of hospitals in the top 25th percentile (ie, highest rates) based on unadjusted cHT-CDI rates to their subsequent ranking using SIRs based on the 2 models.

Analyses were conducted using the Statistical Analysis System version 9.4 software (SAS Institute, Cary, NC).

## Results

The study included 9,134,276 patient admissions (October 2015–March 2020), which were associated with 17,545 cHT-CDI events from 265 acute-care hospitals in the United States. Medical school-affiliated hospitals accounted for 38.1% and urban facilities for 60.8%.

### Summary statistics of cHT-CDI prevalence and bivariate analysis results

The cHT-CDI data exhibited a right-skewed distribution. Over the study period, the median rate of cHT-CDI events per 100 admissions was 0.134 (interquartile range, 0.023–0.243) and the mean rate was 0.166 per 100 admissions (SD, 0.18). We observed evidence of decreases in cHT-CDI event rates during 2015–2022 on bivariate analysis (Table 1). All candidate risk factors considered in the analysis were significantly associated with cHT-CDI, except for 2 age groups (65–80 and >80 years) (Table 1).

### Variables associated with cHT-CDI: Simple model

Using the simple model, we identified the following as significant variables: higher CO-CDI prevalence; longer average LOS; higher rate of ICU admissions; facilities with larger bed size; urban hospitals; and higher percentage of patients aged 41–64 years. In addition, we observed some changes in the cHT-CDI rate over time (Table 2 and Supplementary Table 1 online).

**Table 2.** cHT-CDI–Associated Variables in the Simple Model with Estimated Incidence Rate Ratios (IRRs)^a^

Parameter	IRR (95% CI)^b^	*P* Value
**Year**		
2015	1.44 (1.24–1.68)	<.0001
2016	1.50 (1.33–1.68)	<.0001
2017	1.43 (1.27–1.60)	<.0001
2018	1.24 (1.11–1.39)	.0002
2019	1.05 (0.93–1.17)	.4338
2020		Reference
**CO-CDI prevalence**	2.83 (2.64–3.03)	<.0001
**Average LOS**	1.21 (1.18–1.24)	<.0001
**Bed size**		
1–50		Reference
51–100	1.69 (1.35–2.11)	<.0001
101–200	1.80 (1.46–2.22)	<.0001
201–300	2.33 (1.89–2.87)	<.0001
301–500	2.41 (1.95–2.96)	<.0001
500+	2.19 (1.76–2.71)	<.0001
**% ICU admissions**		
< 2^nd^ quartile		Reference
3^rd^ quartile	1.14 (1.07–1.21)	<.0001
4^th^ quartile	1.18 (1.11–1.26)	<.0001
Not reported	1.46 (1.32–1.62)	<.0001
**% patients aged 41–64 y**	1.01 (1.00–1.01)	0.027
**Urban/rural status**		
Rural		Reference
Urban	1.09 (1.03–1.15)	.0026

Note. CI, confidence interval; CO-CDI, community-onset *C. difficile* infection; cHT-CDI, candidate definition for healthcare facility-onset, treated *C. difficile* infection; ICU, intensive care unit; IRR, incidence rate ratio; LOS, length of stay.

a For model replication purposes, regression coefficients and standard errors are presented in Supplementary Table S1 (online).

b Estimated increase in cHT-CDI relative to the reference. As an example, for bed size 101-200 IRR, 1.80; holding other variables constant in the model, hospitals with bed sizes of 101–200 are expected to have a cHT-CDI rate 1.80 times greater (80% higher) than the hospitals with bed size 1–50.

### Variables associated with cHT-CDI: Complex model

Based on the complex model, the following hospital-level variables were significantly associated with higher cHT-CDI event rates: higher CO-CDI prevalence; fourth quartile of average LOS; larger bed size; increased HO testing intensity, increased HO testing prevalence, and teaching hospitals. Increased CO testing intensity and percentage female patients were negatively associated with cHT-CDI event rates (Table 3 and Supplementary Table 2 online). Among identified variables, HO testing intensity, HO testing prevalence, and CO-CDI prevelance yielded higher incidence rate ratios (IRRs). Comparisons of observed versus predicted events showed that the model was well specified (Supplementary Figs. S1 and S2 online).

**Table 3.** cHT-CDI–Associated Variables Identified in the Complex Model with Estimated Incidence Rate Ratios (IRRs)^a^

Parameter	IRR (95% CI)^b^	*P* Value
**Year**		
Years other than 2017 and 2018		Reference
Year 2017 and 2018	1.08 (1.04–1.12)	.0001
**CO-CDI prevalence**		
1st quartile		Reference
2nd quartile	1.63 (1.52–1.75)	<.0001
3rd quartile	2.33 (2.17–2.51)	<.0001
4th quartile	3.24 (2.99–3.51)	<.0001
**Average LOS**		
≤ 3rd quartile		Reference
4th quartile	1.11 (1.06–1.17)	<.0001
**Bed size**		
1–50		Reference
51–300	1.19 (0.98–1.46)	.0865
301+	1.24 (1.01–1.51)	.0355
**HO testing intensity**		
1st quartile		Reference
2nd quartile	1.45 (1.33–1.58)	<.0001
3rd quartile	1.68 (1.54–1.83)	<.0001
4th quartile	2.03 (1.85–2.23)	<.0001
**CO testing intensity**		
1st quartile		Reference
2nd quartile	0.87 (0.83–0.92)	<.0001
3rd quartile	0.80 (0.75–0.85)	<.0001
4th quartile	0.71 (0.66–0.76)	<.0001
**HO testing prevalence**		
1st quartile		Reference
2nd quartile	1.26 (1.15–1.36)	<.0001
3rd quartile	1.50 (1.38–1.63)	<.0001
4th quartile	1.72 (1.57–1.89)	<.0001
**% Females**		
1st quartile		Reference
2nd quartile	0.92 (0.87–0.97)	.0013
3rd quartile	0.93 (0.87–0.98)	.0089
4th quartile	0.90 (0.85–0.97)	.0032
**Teaching status (medical school affiliation)**		
Nonteaching		Reference
Teaching	1.09 (1.04–1.14)	0.0005

Note. CI, confidence interval; CO, community-onset; CO-CDI, community-onset *C. difficile* infection; HO, hospital-onset; cHT-CDI, candidate definition for healthcare facility-onset, treated *C. difficile* infection; IRR, incidence rate ratio; LOS, length of stay.

a For model replication purposes, regression coefficients and standard errors are presented in Supplementary Table S2 (online).

b Estimated increase in cHT-CDI relative to the reference. For example, for teaching hospitals IRR, 1.09; holding other variables constant in the model, teaching hospitals are expected to have a cHT-CDI rate 1.09 times greater (9% higher) than nonteaching hospitals.

### Comparison of hospital rankings

**Figure 1.**
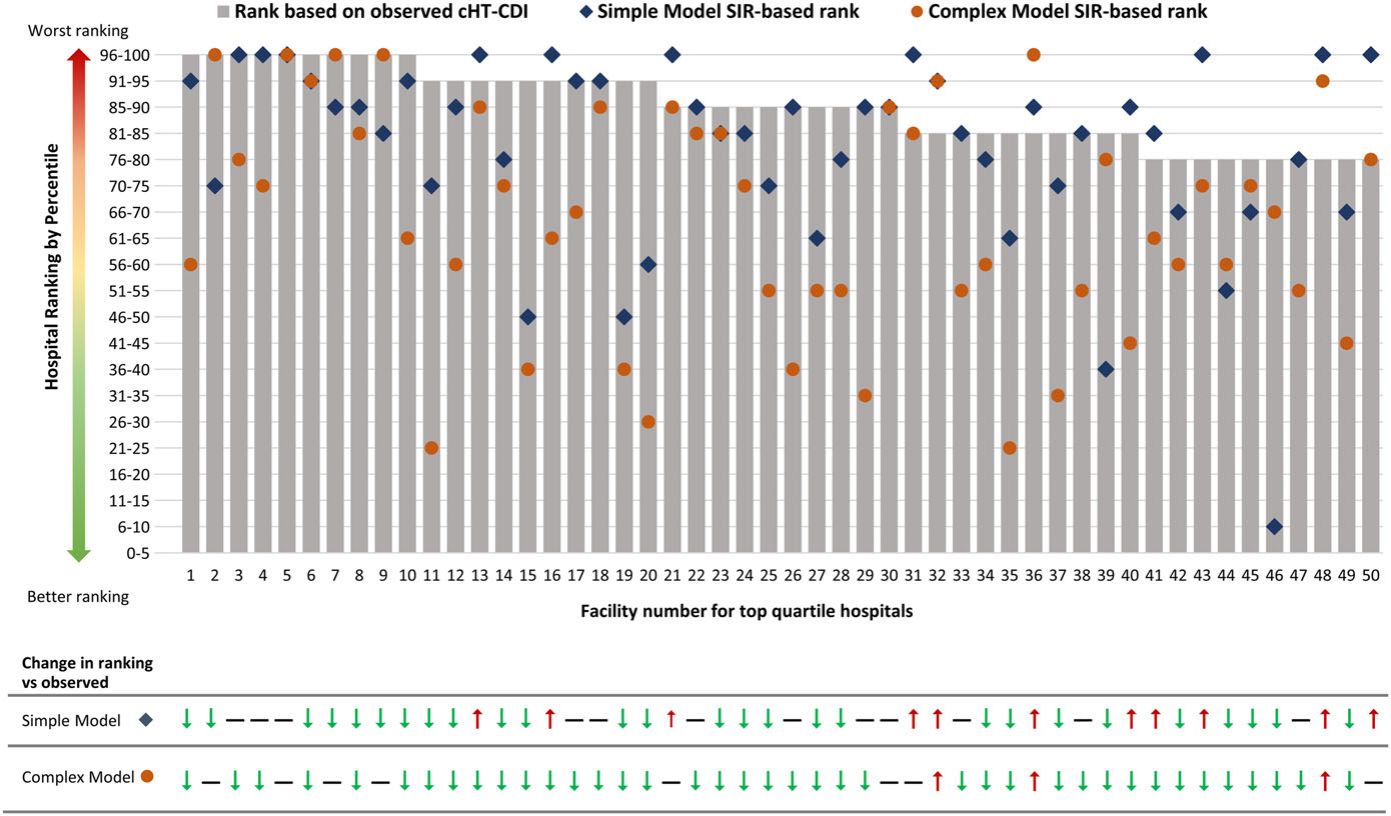
Hospital rankings for top-quartile hospitals (designated 1 through 50) based on observed cHT-CDI rates compared with the simple- and complex-model–derived SIR ranking. Gray bars represent rank of the top quartile of hospitals based on observed cHT-CDI rate per 100 admissions, blue diamonds represent the simple–model SIR-based rank, and orange circles represent the complex–model SIR–based rank. Note. cHT-CDI, candidate definition for healthcare facility-onset, treated *C. difficile* infection; SIR, standardized infection ratio.

We demonstrated a potential real-world application of risk adjustment by quantifying changes in rank of the top 25th percentile (ie, worst-performing) hospitals based on observed cHT-CDI rates compared with complex model–based cHT-CDI SIR ranking. Among the 50 hospitals ranked in the fourth quartile based on unadjusted (observed) cHT-CDI rates, 8 (16%) remained at the same percentile level when using complex model cHT-CDI SIR-based ranking, 39 (78%) improved in percentile ranking, and 3 (6%) shifted to lower (worse-performing) percentiles (Fig. 1). Supplementary Figure S3 shows changes in rank by quartile: 19 (38%) stayed in the same ranking category (fourth quartile) with the complex model–adjusted SIR,^18^ (42%) improved to the third quartile; 8 (16%) improved to the second quartile, and 2 (4%) improved to the first quartile.

Statistical comparison of rankings showed that the unadjusted cHT-CDI rate had a strong ordinal association with the simple model SIR (γ statistic, 0.85; 95% CI, 0.79–0.91). The strength of association between the unadjusted cHT-CDI event rate and complex-model SIR rankings was meaningfully lower (γ statistic, 0.63; 95% CI, 0.52–0.73), thereby demonstrating differential adjustment afforded by the complex model.

## Discussion

Our analysis demonstrates a feasible approach that addresses 2 issues of concern with current HO-CDI reporting: (1) using a more specific and clinically oriented CDI definition based on the combination of a positive CD laboratory test after day 3 of admission plus the use of an anti-CD therapeutic agent and (2) incorporating the impact of testing practices in SIR-adjusted rankings. Our complex-model SIR used current facility- and patient-level risk-adjustment variables, such as bed size, age, sex, and LOS, as well as new testing-practice variables: HO testing intensity, HO testing prevalence, and CO testing intensity (negative correlation). These variables of testing practice have a large influence on cHT-CDI rates, demonstrating their importance to risk adjustment. CO-CDI prevalence was also a strong predictor of cHT-CDI, as evidenced by the ordinal increase of higher IRRs. As described previously,^13^ the simple model was “nested” within the complex model to demonstrate the additional model fit and risk adjustment afforded by SIRs derived by the latter. SIRs, which adjust for facility- and patient-level factors contributing to HAI risk, are used by the CDC and Centers for Medicare & Medicaid Services (CMS) to express performance for various measures of inpatient care, including HAIs.^19–21^ Ultimately, our complex model SIR incorporated a more robust risk adjustment. In total, 39 of 50 hospitals (78%) in the worst-performing quartile moved to lower (better-performing) percentiles, and 31 (62%) hospitals shifted to lower quartiles after complex-model SIR adjustment, including 2 that shifted from the worst- to the best-performing quartile.

The widespread use of PCR in CDI diagnostic testing has raised concerns, particularly the possibility of false-positives due to the high sensitivity and lower specificity of PCR when used alone for CDI diagnosis.^5^ The latest CDI testing and treatment guidelines have attempted to clarify best practices;^1^ however, the resources needed to implement multiple testing tiers or other “reflex” diagnostic rubrics leave strict laboratory test-based CDI definitions at risk for highly variable sensitivity and specificity. In this study, we attempted to address this clinical concern with 2 novel additions to a cHT-CDI metric: (1) linking a laboratory CD test with use of an anti-CD therapeutic agent within a clinically reasonable time frame (the QAD), which may allow the metric to be more reflective of clinically significant CDI and (2) incorporating measures of CD testing practices directly into the complex-model SIR.

We also showed that including measures of CD testing intensity and prevalence in the risk adjustment model may help mitigate concerns about variability^22^ in testing practices within and among facilities. By including HO testing intensity and prevalence, the complex-model risk adjustment may help improve SIR estimation by allowing the resultant expected rate to flex and adjust to changes in testing practices, whether clinically warranted or not. With that in context, the complex model identified HO testing intensity and prevalence as 2 of the most influential variables in cHT-CDI prediction. This makes clinical sense because repeated testing on the same suspected cHT-CDI case, or an increased percentage of patients who receive at least 1 HO-CDI diagnostic test, will have a higher likelihood of yielding a positive cHT-CDI case. This was not the case during the CO period (first 3 days of admission), when CO testing intensity (more testing on a CDI suspect admission) had a significant negative association with cHT-CDI. CO-CDI was also highly correlated with cHT-CDI, suggesting that although community CDI burden does affect cHT-CDI risk, increased testing of random patients within the CO period (testing prevalence) does not. Thus, increasing mass “surveillance” CD testing to mitigate cHT-CDI designations may be unlikely to have an overall net benefit on patient outcomes. Indeed, the resources needed to sustain such testing, if not clinically warranted, are significant and go against the tenets of antimicrobial and diagnostic stewardship.^2^ Therefore, including testing practices in the risk-adjusted model should mitigate attempts to influence SIRs by nonclinical CD testing practices and thus promote best practices.

The finding that higher facility CO-CDI prevalence is correlated with cHT-CDI reinforces central infection prevention tenets of contact precautions and indicates that spore contamination is a significant driver of cHT-CDI,^23,24^ especially for communities with a high CDI burden. These communities likely include more complex patient case mixes, as suggested by the fact that teaching hospital status, LOS, and a higher number of beds also remained significant in the complex model. A recent CMS ruling now requires both infection prevention and control (IPC) programs and antimicrobial stewardship programs (ASPs) as conditions for CMS participation,^25^ and both are important for CDI prevention. Investigators have documented the association between antibiotic use and CDI^26,27^ and have estimated that a 30% decrease in the hospital use of antibiotics linked to CDI could reduce 25% of hospital-associated CDI cases.^26^ Another study reported that an increase of 100 DOT per 1,000 days for high-risk CD agents correlates with a 12% increase in cHT-CDI.^28^ The NHSN Antimicrobial Use and Resistance module^29^ benchmarks antimicrobial use including agents associated with CDI. Furthermore, it is anticipated that a revised cHT-CDI surveillance definition that incorporates anti-CD treatment will also facilitate surveillance for anti-CD treatment (whether CD testing was done or not) using the same data sources. It may become feasible to monitor correlations between the use of antibiotics considered to place patients at high risk for CDI and anti-CD treatment administered without a paired CD test to understand the use of CDI prophylaxis or attempts to avoid registering a cHT-CDI event. Therefore, this analysis not only builds toward a more clinically viable cHT-CDI definition with more robust risk-adjustment but may also provide insights into the relationship between high-risk CDI antimicrobials and therapeutic CDI antimicrobials for both ASP and IPC programs.

This study had several limitations. We did not evaluate individual CDI tests but rather the result of the final CDI test reported by the laboratory because collated EHR data feeds do not always provide visibility as to the sequence of CDI confirmatory tests. Clinical chart review and validation of the cHT-CDI definition were outside the scope of this project. Validation of any new metric against a reference standard of CDI from chart review will need to be evaluated. However, the inclusion of anti-CD agents should improve clinical specificity of a CDI case beyond a mere laboratory designation. Notably, oral metronidazole is used for multiple clinical infections and is not specific to CDI. However, the pairing of an antibiotic requirement with CD testing within 2 days before or after stool collection, and the removal of oral metronidazole as a first line cHT-CDI therapy in more recent CDI guidelines,^1,30^ may minimize this limitation over time. Finally, increasing use of oral vancomycin and fidaxomicin as prophylaxis in high-risk CD-colonized patients^31^ may obscure specificity of cHT-CDI using our metric. It will be important to monitor the use of CDI prophylaxis, delayed treatment, and testing practices, and to leverage those data to adjust the measure in response to changes in clinical practices so that the cHT-CDI definition continues to reflect true infections and incentivize best-care practices.

The risk adjustment achieved with the complex model is distinct and uniquely distinguishes differential changes in cHT-CDI ranking when compared to unadjusted rates. In addition to incorporating factors included in the simple model, the complex model includes differences in CDI testing practices that, in aggregate, improve model fit, may reduce estimation error, and may more accurately reflect underlying patient risk for cHT-CDI than broad facility-level categories. Facility descriptors, patient characteristics, CO-CDI prevalence, and different aspects of CDI testing intensity and prevalence during the HO and CO periods were significant factors associated with cHT-CDI incidence. A national cHT-CDI metric should endeavor to include these characteristics to empower stewardship and infection prevention programs with the highest quality data and thereby reduce CDI and improve patient care.

## Supplementary material

For supplementary material accompanying this paper visit https://doi.org/10.1017/ice.2023.124. click here to view supplementary material
