# Supplementary material for: Treated, hospital-onset Clostridiodes difficile infection: An evaluation of predictors and feasibility of benchmarking comparing 2 risk-adjusted models among 265 hospitals
Source: Infect Control Hosp Epidemiol. 2023 Jul 14;45(1):48–56. doi: 10.1017/ice.2023.124 (PMC10782205; doi:10.1017/ice.2023.124)
Supplement: Supplementary file 1 [file S0899823X23001241sup001.docx]

**Supplementary Materials**

**Treated, hospital-onset *Clostridiodes difficile* infection: an evaluation of predictors and feasibility of benchmarking comparing two risk-adjusted models among 265 hospitals**

Kalvin C. Yu MD^1^, Gang Ye PhD^1^, Jonathan R. Edwards MStat^2^, Raymund Dantes MD, MPH^2,3^, Vikas Gupta PharmD^1^, ChinEn Ai MPH^1^, Kristina Betz MD PhD^2^, Andrea L. Benin MD^2^

^1^Becton, Dickinson and Company, 1 Becton Drive, Franklin Lakes, New Jersey, USA

^2^Centers for Disease Control and Prevention, Atlanta, Georgia, USA

^3^Emory University School of Medicine, Atlanta, Georgia, USA

**Supplementary Table S1.** Regression Coefficients and Standard Errors for the Simple Model.

| **Parameter** | **Regression Coefficient (In Logarithm Scale)** | **Standard Error** |
| --- | --- | --- |
| **Intercept** | -4.450 | 0.137 |
| **Year** |  |  |
| 2015 | 0.366 | 0.079 |
| 2016 | 0.403 | 0.059 |
| 2017 | 0.354 | 0.058 |
| 2018 | 0.218 | 0.058 |
| 2019 | 0.046 | 0.058 |
| 2020 |  |  |
| **COCDI prevalence** | 1.039 | 0.035 |
| **Average LOS** | 0.189 | 0.012 |
| **Bed size** |  |  |
| 1-50 |  |  |
| 51-100 | 0.523 | 0.113 |
| 101-200 | 0.586 | 0.107 |
| 201-300 | 0.844 | 0.107 |
| 301-500 | 0.878 | 0.106 |
| 500+ | 0.783 | 0.110 |
| **% ICU admissions** |  |  |
| < 2^nd^ quartile |  |  |
| 3^rd^ quartile | 0.130 | 0.031 |
| 4^th^ quartile | 0.169 | 0.032 |
| Not reported | 0.379 | 0.053 |
| **% patients aged 41-64 years** | 0.005 | 0.002 |
| **Urban/rural status** |  |  |
| Rural |  |  |
| Urban | 0.083 | 0.028 |

Note. COCDI, community-onset *C.*difficile infection; ICU, intensive care unit; LOS, length of stay.

**Supplementary Table S2.** Regression Coefficients and Standard Errors for the Complex Model.

| **Parameter** | **Regression Coefficient (In Logarithm Scale)** | **Standard Error** |
| --- | --- | --- |
| **Intercept** | -3.370 | 0.111 |
| **Year** |  |  |
| Years other than 2017 and 2018 |  |  |
| Year 2017 and 2018 | 0.078 | 0.020 |
| **COCDI prevalence** |  |  |
| 1^st^ quartile |  |  |
| 2^nd^ quartile | 0.489 | 0.037 |
| 3^rd^ quartile | 0.847 | 0.037 |
| 4^th^ quartile | 1.175 | 0.041 |
| **Average LOS** |  |  |
| ≤ 3^rd^ quartile |  |  |
| 4^th^ quartile | 0.106 | 0.026 |
| **Bed size** |  |  |
| 1-50 |  |  |
| 51-300 | 0.175 | 0.102 |
| 301+ | 0.212 | 0.101 |
| **HO testing intensity** |  |  |
| 1^st^ quartile |  |  |
| 2^nd^ quartile | 0.371 | 0.043 |
| 3^rd^ quartile | 0.516 | 0.045 |
| 4^th^ quartile | 0.709 | 0.048 |
| **CO testing intensity** |  |  |
| 1^st^ quartile |  |  |
| 2^nd^ quartile | -0.136 | 0.028 |
| 3^rd^ quartile | -0.223 | 0.030 |
| 4^th^ quartile | -0.349 | 0.035 |
| **HO testing prevalence** |  |  |
| 1^st^ quartile |  |  |
| 2^nd^ quartile | 0.227 | 0.043 |
| 3^rd^ quartile | 0.403 | 0.043 |
| 4^th^ quartile | 0.544 | 0.047 |
| **% Females** |  |  |
| 1^st^ quartile |  |  |
| 2^nd^ quartile | -0.088 | 0.027 |
| 3^rd^ quartile | -0.077 | 0.029 |
| 4^th^ quartile | -0.100 | 0.034 |
| **Teaching status (medical school affiliation)** |  |  |
| Non-teaching |  |  |
| Teaching | 0.083 | 0.024 |

Note. CO, community-onset; COCDI, community-onset *C.*difficile infection; HO, hospital-onset; LOS, length of stay.

**Supplementary Figure S1.** Goodness-of-fit: Decile Plot of Observed vs Predicted cHT-CDI (HOCDI) events (quarterly average).


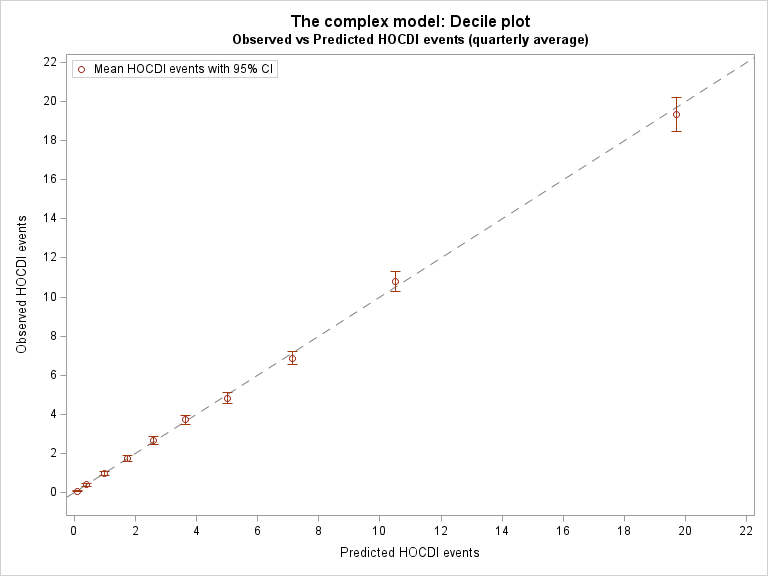


Note: cHT-CDI, candidate definition for healthcare facility-onset, treated *C.*difficile infection; HOCDI, hospital-onset C. difficile infection).

The decile plot shows no systematic pattern of deviation for the model predicted cHT-CDI events from the ‘perfect fit’ (gray dashline), which indicates a well-specified model (i.e., a model that matches the data).

**Supplementary Figure S2.** Goodness-of-fit: Observed vs Predicted cHT-CDI Events (HOCDI) (per Quarter).


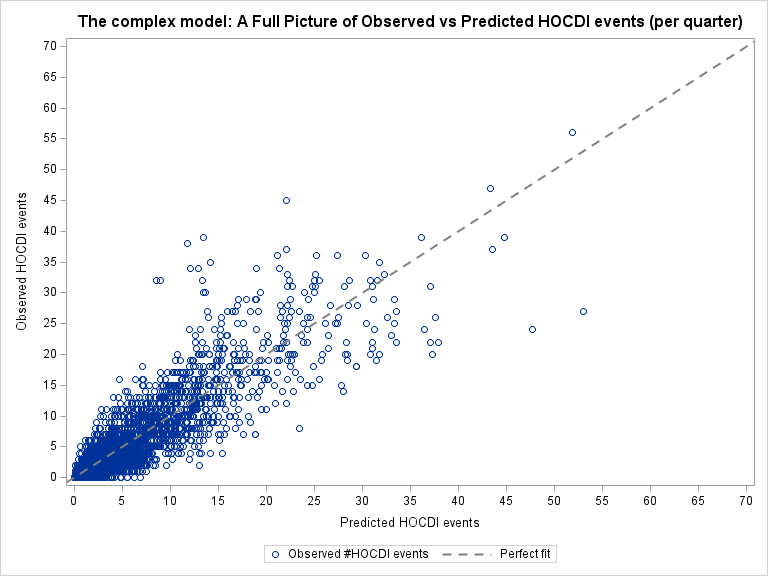


Note: cHT-CDI, candidate definition for healthcare facility-onset, treated *C.*difficile infection; HOCDI, hospital-onset C. difficile infection.

The random distribution of the scattered points of observed vs predicted events along the ‘perfect fit’ line indicates a well-specified model (i.e., a model that matches the data).

**Supplementary Figure S3**. Ranking change of hospitals for the top 25 percentile (4^th^ quartile; worst performing) based on unadjusted observed cHT-CDI event rates (blue triangles) compared to the ranking based on the Complex Model SIR (orange diamonds). A more granular breakdown of these hospitals and the rank adjustment after applying Simple and Complex SIR is included in Figure S1.

Note: cHT-CDI, candidate definition for healthcare facility-onset, treated *C.*difficile infection; SIR, standardized infection ratio.

Among the 50 hospitals ranked in the 4th quartile of the unadjusted (observed) cHT-CDI rate, 19 hospitals (38%) remained in the same ranking category (4th quarter) of model-based (adjusted) SIR and 31 hospitals (62%) improved in ranking when using the Complex Model-based SIR ranking. Specifically, 21 hospitals (42%) improved their ranking to the 3^rd^ quartile; 8 hospitals (16%) improved their ranking to the 2^nd^ quartile; and 2 hospitals (4%) moved to the 1st quartile (from the worst-performing quartile to the best-performing quartile).
